# Influence of hand starting position on radial line bisection

**DOI:** 10.3389/fpsyg.2023.1293624

**Published:** 2023-12-07

**Authors:** Mariateresa Ricci, Alessandro Iavarone, Ciro Rosario Ilardi, Sergio Chieffi

**Affiliations:** ^1^Department of Experimental Medicine, University of Campania “Luigi Vanvitelli”, Naples, Italy; ^2^Neurological Unit, CTO Hospital, AORN “Ospedali dei Colli”, Naples, Italy; ^3^IRCCS SYNLAB SDN, Naples, Italy

**Keywords:** line bisection, radial line, starting position, attention, action

## Abstract

When normal individuals are asked to localize and mark the midpoint of a radial line, they tend to bisect it farther than the true center. It has been suggested that radial misbisection depends on the presence of a visual attentional bias directed toward the far space. The aim of the present study was to investigate whether the localization of the center of radial lines was affected by the starting position of the hand. There were two starting positions: one between the body and the radial line (“near”), the other beyond the radial line (“far”). Thirty-four subjects participated in the experiment. The results showed that (i) participants bisected radial lines farther than the true center, measured with reference to their body, in both near and far condition, and (ii) bisection errors in the near condition were greater than those in the far condition. We suggest that hand starting position and direction of ongoing movement influenced radial line misbisection by modulating visual attentional bias directed to far space.

## Introduction

Line bisection is a visuomotor task widely used in both experimental and clinical settings to explore the allocation of attention along the three dimensions of space. The task requires participants to examine a line, localize its center, and mark it with a pencil stroke.

In neurological clinical practice, line bisection task has been broadly utilized to demonstrate the presence of hemispatial neglect. This syndrome is characterized by an asymmetry in the processing of information in the bodily and/or extrabodily space, contralateral to the brain damage (Heilman et al., 2000; Cubelli, 2017). The patient exhibits deficits in detecting, attending to, or responding to stimuli in the contralesional/neglected space. Hemispatial neglect typically manifests in tasks involving personal and extrapersonal spatial stimuli, but it may also emerge in the exploration and processing of mental images (representational neglect) (Bisiach et al., 1983). A higher prevalence of left spatial neglect following right hemisphere damage has been reported (Heilman et al., 2000; Cubelli, 2017).

In line bisection task, patients with hemispatial neglect typically displace the midpoint of horizontal lines toward the ipsilesional side (Heilman et al., 2000; Cubelli, 2017). Neglect may also occur along radial and vertical dimensions of space. Patients with occipitotemporal lesion may reveal a downward and proximal bias on bisection of vertical and radial lines, respectively (Shelton et al., 1990; Mennemeier et al., 1992); patients with occipitoparietal lesion may show an upward and distal bias on bisection of vertical and radial lines, respectively (Adair et al., 1995; Chieffi et al., 2018a).

Bisection performance has also been investigated in neurologically normal individuals. First, errors to the left of the true center have been reported in bisection of horizontal lines (Bowers and Heilman, 1980). However, other studies did not confirm such a leftward bias (Halligan et al., 1990). Regarding the bisection of radial and vertical lines, usually, normal participants bisect (i) radial lines beyond the true center, with respect to their body, and (ii) vertical lines above the true center (Shelton et al., 1990; Barrett et al., 2002; Chieffi and Ricci, 2002). Similarly, a bias toward the far space has been found by Szpak et al. employing a landmark line bisection task. The authors found that participants perceived the center of the line to be distal to the true center (Szpak et al., 2016).

Shelton et al. attributed radial and vertical misbisection to perceptual/attentional factors. They suggested that attention is biased away from the body (“far peripersonal space”) during visual exploration as the visual system is specialized for detecting distant stimuli (Shelton et al., 1990). The influence of spatiotopic factors in radial line bisection has been confirmed by subsequent studies (Chieffi et al., 2008, 2018b).

One possible explanation for the occurrence of bisection errors due to far attentional bias is that attended stimuli appears magnified compared to unattended ones (Prinzmetal and Wilson, 1997; Masin, 2003). In line with this observation, it is likely that far attentional bias, magnifying the distal and upper portion of radial and vertical lines, moves the location of the subjective midpoint forward and up, respectively.

As we have already mentioned, bisection task is a visuomotor task. Planning of movements directed toward the center of the line requires knowledge of both hand starting position and movement endpoint (i.e., subjective midpoint) (Ilardi et al., 2022a). Some studies have suggested that attention may be biased toward current hand position. This was supported by the observation that the detection of targets presented close to the hand was facilitated compared to the detection of target presented far from the hand (Reed et al., 2006, 2010; Tseng and Bridgeman, 2011). Interestingly, Schendel and Roberstson reported the case of the patient WM, who suffered from a severe left hemianopsia following damage to the right primary visual cortex. The authors observed an improvement in the patient's performance in detecting targets presented in his left “blind” visual field when his left arm was placed into the “blind” field (Schendel and Robertson, 2004). In these studies, it is worth noting that the hand was held in resting state.

Other studies have examined attention allocation during hand movement and found that attention was biased toward the movement target. For example, it has been shown that stimulus detection is enhanced when the stimulus is the target of a reaching movement (Deubel et al., 1998). In their study, Deubel et al., used a dual-task paradigm in which the primary task consisted of performing a reaching movement directed at a cued object. The secondary task required to discriminate between two symbols surrounded by distractors. The results showed that discrimination performance was better when the discrimination stimulus was also the target for manual reaching (Deubel et al., 1998). The same was true when the primary task consisted of a sequence of two or three movements (Baldauf et al., 2006; Baldauf and Deubel, 2008).

In the present study, we examined whether the starting position of the hand with respect to radial lines might influence the localization of the subjective center. Starting position was either between the observer and the line (near hand position) or beyond the line (far hand position). Our predictions were as follows.

(i) In the near hand position, we expected, consistent with previous research, that participants would localize the subjective midpoint beyond the true center.

(ii) In the far hand position, we propose two alternative predictions. The first is that if attention is biased toward the hand starting position, this might enhance far attentional bias related to spatiotopic factors. In this case, bisection errors might be greater than those present in the near hand condition. The second prediction posits that if attention is biased toward the movement endpoint (subjective center), this might attenuate the far spatiotopic attentional bias. Consequently, bisection errors might be smaller than those in the near position condition.

## Methods

### Participants

The participants were students at the University of Campania “Luigi Vanvitelli”. All participants were right-handed and had normal or corrected-to-normal vision. The research was approved by the ethics committee and was performed in line with the 1964 Declaration of Helsinki. Participants provided written informed consent.

### Stimuli

The stimuli were black radial lines drawn and centered on sheets of white paper (29.7 × 21.0 cm). The lines were 2 mm wide and 16, 17, 18, 19, 20, 21, 22, 23, or 24 cm long. Different lengths were used in order to create variability on the stimuli presentation.

### Procedure

**Figure 1:**
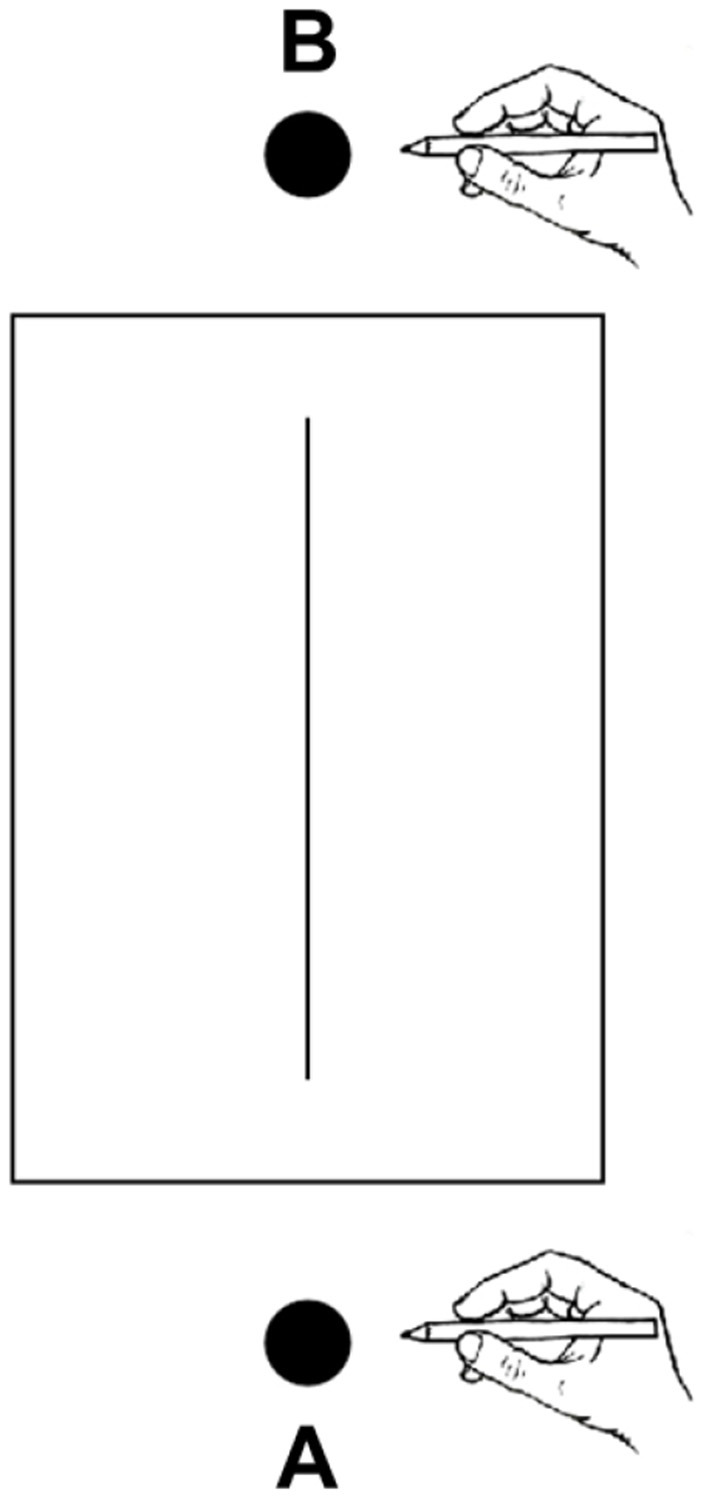
Placement of line bisection stimuli in relation to the starting position of the hand. A, near hand starting position; B, far hand starting position. The proportions of elements are not naturalistic.

The participants were seated at a table (height: 76 cm). Their head was restrained by a chin rest (height: 30.5 cm). Radial lines were presented, one at a time, on the table top at the intersection of the midsagittal and transverse plane. There were two hand starting positions: near and far (see Figure 1). The near position was 5 cm away from the chin rest, and was placed proximally to the line. The far position was placed at 45.5 cm from the chin rest, and was distal to the line. Consequently, the line was located between the two starting positions in such a way that the center of the lines was equidistant from them (20.25 cm). Subjects were required to bisect the radial lines using a pencil held with the right hand. The left hand was placed on legs to avoid any spatial reference during the execution of the task. Participants bisected a total of 144 lines (two starting conditions × nine line lengths × eight trials) administered in two blocks. In one block the starting position of the hand was near, in the other one was far. The order of the two blocks was counterbalanced across subjects. Within each block, line lengths were randomized across trials.

For each stimulus, deviations from the true line center were measured to 0.5 mm accuracy, with negative/positive scores denoting near/far displacements. This measurement was converted to a standardized score, i.e., the percentage deviation score (PDS), using the following formula: (deviation in mm/true line half in mm) × 100 (Facchin et al., 2021). For each condition, we also calculated the variable PDS corresponding to the standard deviation of PDS (VPDS). A variable score is an index of consistency, quantifying the scatter of subjective midpoints.

## Results

To determine if there were differences in the localization of the subjective midpoint between the two hand starting position conditions (near vs. far), we compared PDS values by using a matched-pair *t*-test. Similarly, we investigated if there were differences in performance consistency by comparing VPDS values measured in the two hand starting positions. Furthermore, for each hand starting condition, a one-sample *t*-test was used for comparing PDS with the null set (true center) to examine the direction of misbisection. Cohen's *d* was employed as an effect size estimate and interpreted according to recognized benchmarks, i.e., *d* = 0.2, small effect; *d* = 0.5, medium effect; *d* = 0.8, large effect (Cohen, 2013). Statistical analyses were conducted by means of IMB SPSS Statistics v. 26 and JASP v. 0.16.

G^*^Power 3.1.9.4 was used to perform an a priori power analysis in order to determine the number of participants needed for discerning a minimal effect in the radial line bisection task. The required sample size was computed according to two-tail matched-pair and one-sample *t*-tests. As for the former, at a nominal alpha level (α) of 0.05, power (1 – β) set to 0.80, and minimum average difference on PDS and VPDS fixed at 0.05 cm (SD = 0.10) and 0.10 cm (SD = 0.20), respectively, the required *N* was estimated to be 34. Similarly, 34 sample units were deemed necessary for detecting a minimal difference when contrasting PDS with the null set (α = 0.05, 1 – β = 0.80, H0 = 0.00 cm, H1 = 0.05, *N* = 34).

**Figure 2:**
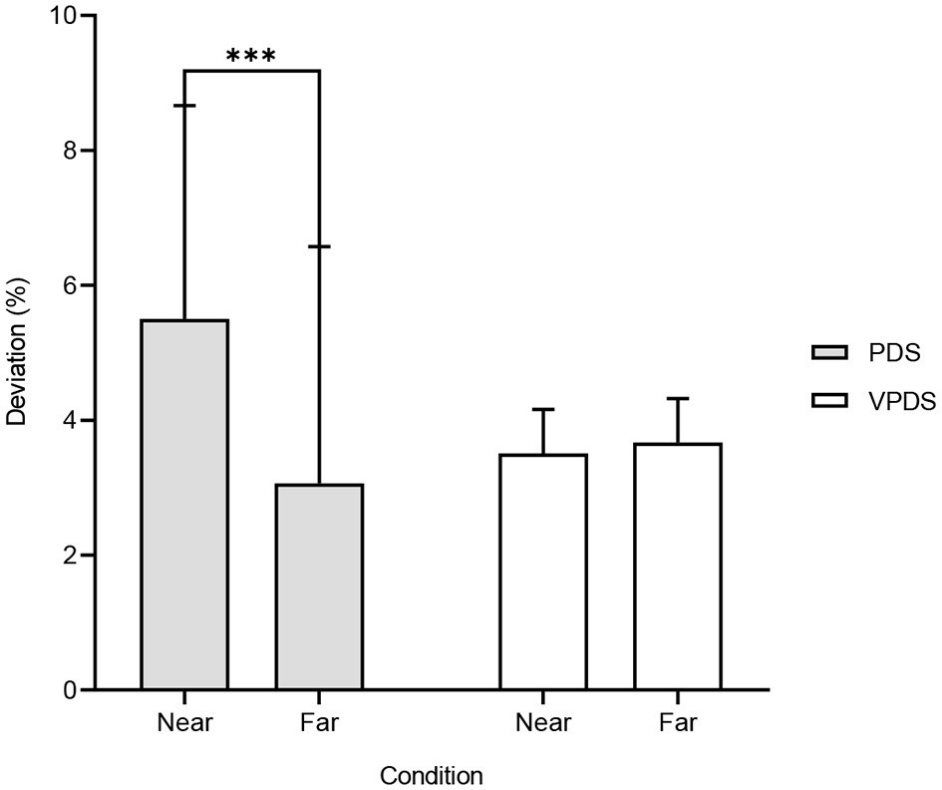
Bar chart depicting the mean percentage deviation for each starting hand position. PDS, percentage deviation score; VDPS, variable percentage deviation score. Error bars represent standard deviation. ***p < 0.001.

Thirty-four participants (13 women and 21 men) took part in this experiment. Their mean age was 24.4 (*SD* = 3.60). Results showed that the starting hand position significantly influenced PDS (*t* = 4.173, *df* = 33, *p* < 0.001; near = 5.50%, *SD* = 3.16 vs. far = 3.06%, *SD* = 3.51; *d* = 0.72). Furthermore, subjects bisected radial lines farther than the true center in both near (*t* = 10.143, *df* = 33, *p* < 0.001, *d* = 1.74) and far (*t* = 5.088, *df* = 33, *p* < 0.001, *d* = 0.87) condition. Conversely, VPDS was not affected by the hand starting position (*t* = −1.735, *df* = 33, *p* = 0.09; near = 3.50%, *SD* = 0.65 vs. far = 3.67%, *SD* = 0.65). Results were plotted and displayed in Figure 2.

## Discussion

The current study aimed at investigating if hand starting position affected the localization of the subjective midpoint in a radial line bisection task. Our findings showed that participants bisected radial lines farther than the true center both when the hand starting position was between the line and their body, and when it was beyond the line.

As mentioned in the introduction, it has been hypothesized that misbisection of radial lines might depend on the presence of a spatial attentional bias toward the far space. According to this hypothesis, such an attentional bias would shift the location of the subjective midpoint forward, i.e., beyond the true center. However, other authors have suggested that radial lines misbisection might depend on retinotopic factors (Geldmacher and Heilman, 1994). This hypothesis stems from the observation that the distal portion of the line is projected onto the lower hemiretina, which is specialized for visual search and recognition mechanisms directed toward the far space (Previc, 2011). In their experiment, Geldmacher and Heilman asked participants to bisect radial lines presented either below or above the eye level. In the latter condition, there was a conflict between spatial and visual factors, as the proximal portion of the line was projected onto the lower hemiretina. The authors observed that, in the above condition, the error did not differ from zero. Thus, they concluded that both spatial and visual factors played a role in the bisection of radial lines (Geldmacher and Heilman, 1994). In a subsequent study, no effect of retinotopic factors was found (Chieffi et al., 2008). Particularly, participants were required to bisect radial lines placed near to, or far from, the observer. Note that as the stimulus distance increases, the ratio between the visual angle subtended by the distal portion to that subtended by the proximal portion of the lines decreases. The results showed that participants bisected the lines presented in the far space farther than those presented in the near space, according to a spatiotopic processing scheme (Chieffi et al., 2008). Finally, as observed by one of the anonymous referees of this paper, an interesting alternative hypothesis is also possible. As radial lines are presented below the eye level, the visual angle would make the proximal portion to appear larger/longer, and the distal portion to appear smaller/shorter. The misbisection persistently seen in the present study, and past studies, might arise from participants overcorrecting for the vanishing point effect.

In our study, although the subjective center was located beyond the true center for both near and far starting hand conditions, the error was greater in the near than in the far condition. As concerns the latter finding, one possible explanation is that hand movement direction toward the subjective center was opposite to, and interfered with the direction of far attentional bias.

Line bisection is a visuomotor task in which allocentric and egocentric computations would take place (Ilardi et al., 2021, 2022b). At first, the localization of the subjective midpoint would require an allocentric estimation as the line is divided in two segments whose magnitude is compared. Comparing two objects is assumed to be an allocentric task (Suavansri et al., 2012). Once the subjective center has been identified, a reaching movement directed toward it has to be programmed. In movement planning, knowledge of both the starting hand position and movement endpoint is essential. Furthermore, experimental evidence suggests that attention is primarily deployed between the starting hand position and the target location (Tipper et al., 1992). In a seminal study, Tipper et al. investigated how the presence of a distractor might interfere with the planning and execution of pointing movements directed to a target. They observed that the distractor interfered with the movement only when it was located within the space between the starting hand position and the target location (Tipper et al., 1992). Other studies have also suggested that, during reaching movements, the attentional focus is biased toward target locations. Deubel et al. found that when participants prepare a pointing movement to a location, visual attention is deployed to the goal position already before movement onset (Deubel et al., 1998). Similarly, Festman et al. observed that when participants simultaneously performed continuous hand motion and a visual discrimination task, discrimination performance was better when the probe was presented at the movement's end location rather than at its start location. Further, there was a direction effect: discrimination performance was better when the hand moved toward the visual probe (Festman et al., 2013a,b).

The close interaction between action and attention agrees with the overarching concept of the “premotor theory” of attention, as proposed by Rizzolatti et al. According to the “premotor theory”, there exists a degree of overlap between the motor and spatial attention control systems. Furthermore, when a movement directed to a target is programmed, attention would shift toward the target's position in space (Rizzolatti et al., 1994).

Concerning our experiment, it is plausible that when the starting hand position was near, and the movement direction proceeded from near to far, the direction of attentional bias related to the ongoing movement was congruent with that of attentional bias directed toward far space. Both attentional biases might contribute to shifting the location of the subjective center beyond the true center, in relation to the participant's body. Conversely, in the far hand condition, the direction of ongoing movement was from far to near. In this case, the direction of attention bias related to the ongoing movement was opposite to that of attentional bias toward far peripersonal space. This incongruence might have resulted in a reduction of the strength of the attentional bias toward distant space and, consequently, a decreasing in the bisection error.

Interestingly, VPDS did not differ between the two hand starting conditions. Variable scores quantify the scatter of subjective midpoints and are sensitive to variability or inconsistency in responding. Therefore, in our experiment, the consistency of bisection performance was similar in the near and far starting position of the hand.

In our study, line bisection was performed under binocular viewing condition. Binocular vision allows three-dimensional perception through the process of stereopsis, which refers to the ability to see depth based on the disparity of the two retinal images (Coren et al., 2013). Conversely, monocular vision does not provide stereopsis. It uses some visual cues to judge depth and distance, e.g., linear perspective, relative object size, overlap (or occlusion), and experiential factors (Coren et al., 2013). It has been suggested that monocular vision is associated with a preferential activation of attentional systems in the contralateral hemisphere, and that right hemisphere is biased toward far space (Roth et al., 2002). In a previous study, Roth et al. investigated the effect of monocular viewing on bisection performance in both right- and left-eye-dominant individuals. The results showed that both groups localized the subjective center beyond the true center. However, bisection errors were greater when right-eye dominant individuals used the left eye and left-eye dominant individuals used the right eye. The authors proposed that right-eye-dominant individuals have a hemispheric organization for spatial attention to near and far space, which is the opposite of that in left-eye-dominant subjects (Roth et al., 2002).

One limitation of the current study was the number of participants, which met the minimum threshold required to detect a subtle effect. Consequently, increasing the sample size could enhance the results reliability. In addition, the experiment was conducted under stereoscopic vision. A potential development of the current research might involve comparing performance under binocular vs. monocular vision conditions. Monocular vision is expected to compromise accuracy in depth perception and line length estimation compared to binocular vision. This, in turn, might affect both the subjective midpoint localization (PDS) and the consistency of performance (VPDS).

In conclusion, the observations of the present experiment suggest the existence of a close interaction between attentional factors related to spatial exploration and ongoing movement. Such interaction might modulate the localization of the subjective midpoint of lines oriented in the radial dimension of space.

## Data availability statement

The raw data supporting the conclusions of this article will be made available by the authors, without undue reservation.

## Ethics statement

The studies involving humans were approved by Ethics Committee, University of Campania “Luigi Vanvitelli”. The studies were conducted in accordance with the local legislation and institutional requirements. The participants provided their written informed consent to participate in this study. Written informed consent was obtained from the individual(s) for the publication of any potentially identifiable images or data included in this article.

## Author contributions

MR: Conceptualization, Investigation, Writing – original draft. AI: Conceptualization, Investigation, Writing – original draft. CRI: Writing – original draft, Writing – review & editing, Methodology, Formal analysis. SC: Conceptualization, Investigation, Writing – original draft.
